# Out of Asia: feline stool-associated circular DNA virus (FeSCV) in Brazilian domestic cats

**DOI:** 10.1007/s00705-026-06576-7

**Published:** 2026-04-02

**Authors:** Luiza Saad Pierucci, Raquel Gomes Catozo, Livia Lissa Kobayashi, Sheila Oliveira de Souza Silva, Aline Santana da Hora, Paulo Eduardo Brandão, Sueli Akemi Taniwaki

**Affiliations:** 1 Department of Preventive Veterinary Medicine and Animal Health, School of Veterinary Medicine and Animal Science, University of São Paulo, São Paulo, São Paulo, Brazil; 2 Institute of Tropical Medicine of São Paulo, School of Medicine, University of São Paulo, São Paulo, São Paulo, Brazil; 3 Veterinary Etiological Research Laboratory, School of Veterinary Medicine and Animal Science, Federal University of Uberlândia, Uberlândia, Minas Gerais Brazil

## Abstract

Feline stool-associated circular DNA virus (FeSCV), was identified in Japan (2018) and China (2021) but has not yet been detected outside Asia. In this study, 137 fecal or rectal swab samples from 83 cats were screened for circoviruses and FeSCV, with detection of two putative novel *Cyclovirus* species and 19.3% (16/83) of FeSCV. A higher incidence of FeSCV in kittens and young adults, and FCoV-positive cats with clinical signs of feline infectious peritonitis were observed. Further investigation will clarify the virus pathogenicity and its association with other pathogens.

**Supplementary Information:**

The online version contains supplementary material available at 10.1007/s00705-026-06576-7.

In the past decade, new circoviruses have been found in humans, bobcats, giant pandas, and domestic canines and felines, among other species [1–5]. Circoviruses are grouped into the order *Cirlivirales*, consisting of circular viruses (15–25 nm in diameter) with positive-sense single-stranded DNA of 1.7–3.8 kb. Within this order, the family *Circoviridae* is divided in two genera: *Circovirus*, which are found in mammals, birds and fish; and *Cyclovirus*, which are detected in vertebrates and invertebrates [6]. In domestic cats, two circoviruses have been described: feline stool-associated circular DNA virus (FeSCV) [7] and feline-associated cyclovirus (FeCV) [8].

First identification of FeSCV occurred in 2018 in diarrheic and healthy cats from the same cattery [7]. Phylogenetic analysis showed that it didn’t belong to the genus *Circovirus* nor *Cyclovirus*, and was considered an unclassified circular DNA virus [7, 9]. Recently, FeSCV was classified in the ICTV as phylum *Cressdnaviricota*, class *Arfiviricetes*, order *Cirlivirales*, family *Vilyaviridae*, genus *Andurilvirus*, species *Andurilvirus erebor* [6].

In 2014, FeCV (order *Cirlivirales*, family *Circoviridae*, genus *Cyclovirus*, species *Cyclovirus gato*) was identified in a virome study of domestic cat feces in the United States [8]. However, there was no data on its pathogenesis or prevalence. In this paper we report the first description of FeSCV in Brazil and two novel cycloviruses detected in cats’ fecal samples. We also analyzed risk factors associated with FeSCV infection.

DNA samples (137) from rectal swabs or stool from 83 cats (Supplementary Information 1) were analyzed. Out of these samples, 29 were from single sampling and 108 were serial sampling taken from 54 cats, 3 to 12 months apart from each other. Cats were classified by age according to the American Association of Feline Practitioners (AAFP) and American Animal Hospital Association (AAHA) guidelines (2021) [10]. Previous testing for feline immunodeficiency virus (FIV - *Lentivirus felimdef*) and feline leukemia virus (FeLV - *Gammaretrovirus felleu*) was carried out in 87.9% (73/83) of the cats using SNAP FIV/FeLV combo test (IDEXX), and 96.4% (80/83) of rectal swabs were tested for feline coronavirus (FCoV - *Alphacoronavirus suis*) with RT-qPCR targeting the E gene.

A clarification protocol with three cycles of freezing/thawing were performed in all samples, prior to DNA extraction with RiboPure™ RNA Purification kit (Ambion^®^), in 123 swab samples from a previous research project, or PureLink™ Genomic DNA Mini Kit (Invitrogen) in 14 fecal samples, as recommended by the manufacturers. The extracted DNA was stored at − 20 °C until use. The integrity of nucleic acids was confirmed by 16S gene PCR [11], with 1× DreamTaq Green PCR Master Mix (ThermoFisher Scientific), 400 nM of primers 519A (sense) and 907B (antisense) and 2 µL of extracted DNA, in a 20 µL reaction. Amplification conditions were: 94 °C/5 min; 35 cycles of 94 °C/30 s, 50 °C/30 s and 72 °C/30 s; and 72 °C/5 min. Positive and negative control were, respectively, DNA from a culture of *Leptospira interrogans* serovar Canicola and ultrapure water.

Samples were screened for circoviruses using a hemi-nested PCR targeting the Rep gene [1]. The 20 µL reaction contained 1× DreamTaq Green PCR Master Mix (ThermoFisher Scientific), 500 nM of each primer pair (CV-F1/CV-R1 or CV-F1/CV-R2) and 2 µL of extracted DNA or 1 µL of PCR product, respectively for the PCR and hemi-nested PCR reaction. The PCR conditions were: 94 °C/5 min; 14 cycles of 94 °C/30 s, 48 °C/30 s (with an increase of 0.5 °C per cycle) and 72 °C/1 min s; 31 cycles of 94 °C/30 s, 55 °C/30 s and 72 °C/1 min; and 72 °C/5 min; and hemi-nested PCR were: 94 °C/5 min; 40 cycles of 94 °C/20 s, 58 °C/20 s and 72 °C/45 s and 72 °C/5 min. Positive and negative controls were PCV-2 DNA sample and ultrapure water, respectively.

After, samples were tested with specific primers for FeSCV targeting the Rep gene [7], with some modifications: FeSCV1F-alt (5’ GCT AAG GTC TGG CTC AG 3’) and FeSCV1R-alt (5’ CTA TRT CCA GGT CGG GAG 3’). PCR used a total volume of 20 µL, with 1× DreamTaq Green PCR Master Mix (ThermoFisher Scientific), 400 nM of each primer, and 2 µL of extracted DNA. Amplification conditions were: 94 °C/5 min; 50 cycles of 94 °C/20 s, 53 °C/20 s and 72 °C/30 s and 72 °C/5 min. A positive sample from hemi-nested PCR and ultrapure water were used as positive and negative controls.

All amplicons were analyzed by electrophoresis in a 1.5% agarose gel stained with SYBR^®^ Safe DNA gel Stain (Invitrogen™) compared with GeneRuler 100 bp DNA ladder (Thermo Scientific™), and were purified with PureLink™ Quick Gel Extraction Kit (Invitrogen) or ExoSAP-IT Express PCR Product Cleanup Reagent (ThermoFisher Scientific™), as the manufacturer’s instructions. Bidirectional sequencing was performed by BigDye Terminator v3.1 Cycle Sequencing Kit (Applied Biosystems), using EDTA and ethanol purification protocol, and the ABI 3500 sequencer (Applied Biosystems), following manufacturer’s recommendations. The reliability of resulting sequences was analyzed with Phred Electropherogram Quality Analysis software [12], checked and edited manually on MEGA X software [13], and the consensus sequences were assembled with Cap-Contig program available in Bioedit software [14].

Seven sequences were aligned with GenBank database reference sequences of FeCV, FeSCV and other cycloviruses, circoviruses and circular Rep-encoding ssDNA virus (CRESS DNA virus), using codon alignment with Muscle tool implemented in MEGA X software [13]. The unrooted nucleotide phylogenetic tree was constructed using Maximum Likelihood (ML) method and Tamura-Nei 93 (TN93) evolutionary model, with 1,000 replicates (bootstrap), gamma distribution and invariable sites [13]. The nucleotide and amino acid identity of sequences were calculated using Bioedit software [14].

The association of FeSCV infection regarding risk factors, such as age, sex, breed, coinfection with FIV, FeLV, FCoV with and without sign of feline infectious peritonitis (FIP) were statistically analyzed using Fisher’s exact test and Qui-square ran by R v4.5.

Eight cats (9.6% − 8/83) were positive for circoviruses hemi-nested PCR, but only seven sequences were submitted to phylogenetic analyses (Fig. 1). Sample 6 shared an identity of 97.8% for nucleotides (nt) and 98.3% for amino acids (aa) with FeSCV KU14 (NC040381). Sequences from samples 32, 34, 36, 38 and 96 (SP7 cattery) were tightly related to each other (99.7% − 100% nt and 100% aa identity) and segregated with bat-associated cyclovirus (*Cyclovirus bastao*, isolate POA/2012/VI - NC025792), showing 95.6–95.9% nt and 93.9% aa of identity. Despite this virus being clustered with bat associated cyclovirus, found in insectivorous bats from South Brazil [15], it has a low amino acid identity with our samples, which could suggest a new viral species. Sample 54 segregated with army ant cycloviru*s* (*Cyclovirus fourmi* - ON324073), with identity of 98.2% (nt) and 100% (aa). They may be feline specific viruses or a species hosted by insects, as cats hunt these animals and cycloviruses are likely to have them as hosts. Regarding FeCV (NC024700), all sequences from this study had low identity, with only 39.2–48.1% (nt) and 32.5–41.5% (aa) of identity, which shows that they are not the same virus.

**Fig. 1:**
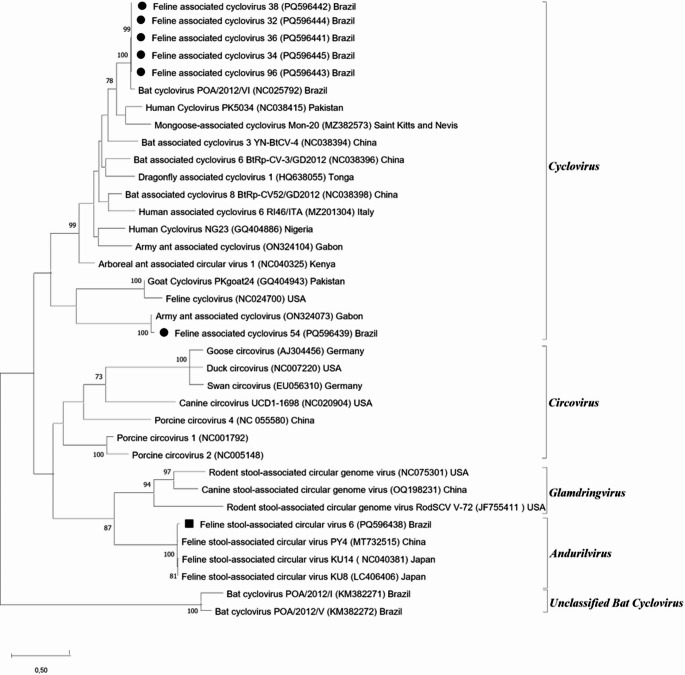
Nucleotide phylogenetic tree (281 nt) of the Rep gene with 40 representative sequences of *Cirlivirales* order, constructed with Maximum Likelihood (ML) method and Tamura-Nei (TN93) model with gamma distribution (5 categories) and evolutionary invariable sites (I). Bootstrap values (1,000 replicates) above 70 are shown in nodes and bars represent the number of substitutions per site. Circles and square indicate *Cyclovirus* and FeSCV from this study, respectively. GenBank accession numbers are shown in parentheses

Concerning the risk factors associated with circoviruses (Table 1), females (15.4% − 8/52) were more likely to be positive (*p*-value = 0.02275). However, the study had more females than males. Most of the *Cyclovirus* positive cats were breeding animals, who frequently experienced heat, gestation and lactation, which can be stressful and could lead to immunosuppression. Besides, cattery SP7, where the majority of the felines positive for *Cyclovirus* were housed (5/7), had inadequate welfare and sanitary conditions, another contributing factor to increased stress.

When screening for FeSCV specifically, 19.3% (16/83) of the cats were positive. Fifty samples were confirmed by genetic sequencing, with identity of 98.1 to 100.0% (nt) and 97.7 to 100% (aa). Only one cat remained positive in the second collection 10 months later, while all other cats showed positive results only at first or second sampling. Analysis of different risk factors (Table 1), showed that regarding age, young adults and kittens together (14/52–26.9%), were more likely to be positive to FeSCV (*p*-value = 0.00912). Young cats tend to interact more with other cats, thus they would be more prone to virus exposure, furthermore young animals are developing immune responses to most pathogens, which could make them more susceptible when exposed to pathogens.

**Table 1:** Risk factors associated with circoviruses and FeSCV

Risk factors		Number of animals	Circoviruses			FeSCV		
			Positive	Negative	*p*-value	Positive	Negative	*p*-value
Sex	Females	52 (62.6%)	8 (15.4%)	44 (84.6%)	0.02275^*^	8 (15.4%)	44 (84.6%)	0.2443
	Males	31 (37.4%)	0	31 (100%)		8 (25.8%)	23 (74.2%)	
Age	Kitten	14 (16.9%)	2 (14.3%)	12 (85.7%)	0.2479	6 (42.9%)	8 (57.1%)	0.00912^**,a^
	Young Adult	38 (45.8%)	5 (13.2%)	33 (86.8%)		8 (21.0%)	30 (78.9%)	
	Mature Adult	13 (15.7%)	0	13 (100%)		1 (7.7%)	12 (92.3%)	
	Senior	16 (19.3%)	1 (6.3%)	15 (93.7%)		0	16 (100%)	
	No data	2 (2.4%)	0	2 (100%)		1 (50.0%)	1 (50.0%)	
Breed	DSH	52 (62.6%)	3 (5.8%)	49 (94.2%)	0.2271	8 (15.4%)	44 (84.6%)	0.1991^b^
	Persian	17 (20.5%)	4 (23.5%)	13 (76.5%)		5 (29.4%)	12 (70.6%)	
	Maine Coon	10 (12%)	1 (10%)	9 (90%)		3 (30.0%)	7 (70.0%)	
	Birman	1 (1.2%)	0	1 (100%)		0	1 (100%)	
	Siamese	3 (3.6%)	0	2 (100%)		0	3 (100%)	
Total		83	8	75		16	67	

*DSH* domestic shorthair;

statistical significance: ^*^p < 0.05; ^**^p < 0.01

^a^Comparing young adult and kitten together with mature adult and senior together

^b^Comparing DSH with all the breeds combined

No cats were FIV positive and 5.5% (4/73) were FeLV positive. Out of the 80 cats tested for FCoV, 52 (65.0%) were positive and 28 (35.0%) were negative. One cat was positive for feline associated cyclovirus and FeSCV at the same time, and only one cat tested positive for FeLV, FCoV and FeSCV at first sampling. This patient was retested after four months and remained positive for FeLV and FCoV but tested negative for FeSCV. Due to a large number of animals showing different results for FCoV and FeSCV between the first and second sampling, the statistical analysis was carried out with a total number of samples (134), instead of the number of cats (80). Among FeSCV positive samples, 58.8% (10/17) were also FCoV positive (Table 2). Out of those, 50% (5/10) showed clinical signs of FIP, which was statistically relevant (*p*-value = 0.006861). The majority of the circovirus positive samples (6/8–75.0%) were FCoV positive, but it was not statistically significant. It was not possible to associate risk factors with retroviruses since only 4 cats were positive for FeLV and none for FIV.

**Table 2:** Association between FeSCV and FCoV

FCoV		Number of samples	Circoviruses			FeSCV		
			Positive	Negative	*p*-value	Positive	Negative	*p*-value
FCoV +	with FIP	8	1 (12.5%)	7 (87.5%)	0.1618	5 (62.5%)^*^	3 (37.5%)	0.3983
	w/o FIP	58	5 (8.6%)	53 (91.4%)		5 (8.6%)	53 (91.4%)	
FCoV-		68	2 (2.9%)	66 (97.1%)		7 (10.3%)	61 (89.7%)	
Total		134	8 (6.0%)	126 (94.0%)		17 (12.7%)	117 (87.3%)	

^*^p-value = 0.006861, comparing FCoV positive samples with and without sign of FIP

This study shows the occurrence of FeSCV in feline stool outside of Asia, a previously unknown fact. In Japan, FeSCV was identified in 65.0% (13/20) of fecal samples from domestic cats by PCR with specific FeSCV primers. Among the positives, 76.9% (10/13) were diarrheic and 23.1% (3/13) were healthy [7]. Similar to our study, the circoviruses hemi-nested PCR screening had less sensitivity, so, it is essential to perform the specific PCR for FeSCV. In China, Hao and colleagues [9] found FeSCV in 20.0% (2/10) of samples from diarrheic cats with the FeSCV specific primer, similar to the occurrence in our study (19.3%), but unfortunately, we did not have information about stool consistency.

Due to small sample sizes and scarcity of studies, there is still not enough evidence to determine FeSCV pathogenicity. Both studies suggest it is possibly related to enteric diseases in domestic cats, especially in co-infection with enteric viruses, such as FCoV, Feline Panleukopenia Virus, Feline Astrovirus, Feline Kobuvirus and Feline Bocavirus [8, 9]. In our study there was no statistical association with co-infection with FeSCV and FCoV, despite 58.8% (10/17) of FeSCV positive samples being also positive for FCoV, with half of them having FIP signs. This indicates that animals with FCoV, especially those with signs of FIP, may be more susceptible to FeSCV infection (*p*-value = 0.006861). Unfortunately, it was not possible to verify the correlation with retrovirus infection, which is highly related to decreased cat immunity, as there were no FIV and FeLV animals in the sample in significant numbers.

FeSCV-positive cats from the same cattery were diagnosed in the same sampling. In SP7 cattery, all positive results (4/10) were on the second sampling. During the interval of sampling (12 months), 4 new cats were introduced into this cattery, and it is possible that one of these animals introduced the virus to the group, but there were no samples of these cats. In this cattery, sample 38 was FeSCV and feline associated cyclovirus positive at the same time. Felines from the catteries SP1 (3/10) and RJ (2/19) were positive in the first collection, only one of them was still positive on the second sampling. This cat was classified as kitten and was FCoV-positive (without FIP) and maintained the positive result for 10 months. All other 5 kittens positive for FeSCV, that were FCoV-positive (with FIP), had only one sampling, including 4/5 cats from Jundiaí cattery (Supplemental Data 1). This suggested a transient FeSCV infection, with long-term persistence in kittens. This study as well as Takano and colleagues [7] sampled cats from catteries. Enclosed spaces with multiple animals, such as catteries, may favour dissemination of viruses, as the animals are in closer contact with each other.

Although two novel cycloviruses were found, the sequenced fragments were small (341–500 nt), therefore, it would be necessary to sequence the entire virus genome to determine the species precisely. Other *Cressdnaviricota* species have already been described in domestic felines, such as canine circovirus (species *Circovirus canine*) [6]. In wild felids, a new species of *Circovirus* was identified in stool samples of *Lynx rufus* (Bobcat). The study was carried out in Mexico and obtained 13.6% (3/22) of positive samples [2]. Susceptibility to circoviruses in phylogenetic close species has already been observed by the detection of PCV type 3 in 10.0% (7/70) of serum samples from free-ranging wild boars in Brazil [16]. Further investigation of *Cressdnaviricota* viruses in domestic cats and wild felids, such as epidemiological data on viral distribution and development of clinical signs, and associate the co-infection with other feline viruses, and analysis of the complete genome is required for phylogenetic classification and for a better understanding of the risks to animal and human health.

## Supplementary Information

Below is the link to the electronic supplementary material.

Supplementary Material 1

## Data Availability

Nucleotide sequences of this study have been deposited in the GenBank database (PQ596438-PQ596445; PX738468-PX738481).
